# When DNA Tells the Tale: High-Resolution Melting as a Forensic Tool for Mediterranean Cetacean Identification

**DOI:** 10.3390/ijms26157517

**Published:** 2025-08-04

**Authors:** Mariangela Norcia, Alessia Illiano, Barbara Mussi, Fabio Di Nocera, Emanuele Esposito, Anna Di Cosmo, Domenico Fulgione, Valeria Maselli

**Affiliations:** 1 Department of Biology, University of Napoli Federico II, Via Cinthia 26, 80126 Napoli, Italy; mariangela.norcia@unina.it (M.N.); ale.illiano19@gmail.com (A.I.); dicosmo@unina.it (A.D.C.); fulgione@unina.it (D.F.); 2 Oceanomare Delphis APS, Viale Rimembranze 14, 47924 Rimini, Italy; 3 Istituto Zooprofilattico Sperimentale del Mezzogiorno, Via Salute, 2, Portici, 80055 Naples, Italy; fabio.dinocera@izsmportici.it (F.D.N.); emanuele.esposito@izsmportici.it (E.E.); 4 MNESYS—PNRR Partenariato Esteso, 16132 Genova, Italy

**Keywords:** High-Resolution Melting analysis, cetacean species identification, species assignment, *Tursiops truncatus*, *Stenella coeruleoalba*, *Physeter macrocephalus*, *Balaenoptera physalus*, cetacean DNA genotyping

## Abstract

Effective species identification is crucial for the conservation and management of marine mammals, particularly in regions such as the Mediterranean Sea, where several cetacean populations are endangered or vulnerable. In this study, we developed and validated a High-Resolution Melting (HRM) analysis protocol for the rapid, cost-effective, and reliable identification of the four representative marine cetacean species that occur in the Mediterranean Sea: the bottlenose dolphin (*Tursiops truncatus*), the striped dolphin (*Stenella coeruleoalba*), the sperm whale (*Physeter macrocephalus*), and the fin whale (*Balaenoptera physalus*). Species-specific primers targeting mitochondrial DNA regions (cytochrome b and D-loop) were designed to generate distinct melting profiles. The protocol was tested on both tissue and fecal samples, demonstrating high sensitivity, reproducibility, and discrimination power. The results confirmed the robustness of the method, with melting curve profiles clearly distinguishing the target species and achieving a success rate > 95% in identifying unknown samples. The use of HRM offers several advantages over traditional sequencing methods, including reduced cost, speed, portability, and suitability for degraded samples, such as those from the stranded individuals. This approach provides a valuable tool for non-invasive genetic surveys and real-time species monitoring, contributing to more effective conservation strategies for cetaceans and enforcement of regulations against illegal trade.

## 1. Introduction

The Mediterranean Sea hosts a remarkably diverse cetacean fauna, with a total of twenty-four species documented as currently or historically visiting the region, although it represents a relatively small portion of the world’s oceans [1,2]. Without considering the contiguous Atlantic area, the species currently recognized as regular in the Mediterranean, and well adapted to the region’s environmental conditions, are nine [3], with the rough-toothed dolphin (*Steno bredanensis*) present only in the Levantine Sea. All these regular species are represented in the Mediterranean by populations genetically distinct from their North Atlantic relatives. Fifteen other species occur or have occurred in the Mediterranean as vagrants from adjacent regions [3].

Among the regular species of dolphins, the most frequently recorded in the Mediterranean Sea are the striped dolphin (*Stenella coeruleoalba*) and the common bottlenose dolphin (*Tursiops truncatus*) [1,4,5,6,7]. These species exhibit high relative abundance and encounter rates across multiple monitoring programs, reflecting both their ecological adaptability to Mediterranean environmental conditions and their role as key components of the region’s marine ecosystems. Both species were downgraded in the last International Union for Conservation (IUCN) assessment [8], from Vulnerable to Least Concern.

The striped dolphin, which was previously defined as Vulnerable under criterion A2bcde due to the decline of mature individuals subject to threats such as bycatch, marine traffic, and ship strikes [9], is now listed as Least Concern thanks to recent data obtained from the ACCOBAMS Survey Initiative (ASI), which evaluated the Mediterranean subpopulation of striped dolphins as significantly larger and increasing [10]. The Mediterranean subpopulation of the common bottlenose dolphin was the subject of historical intentional killing and suffered from habitat degradation and overfishing of prey [11,12,13]. In 2012, their decline led to the assessment of the species as Vulnerable under criterion A2cde, implying a population drop of over 30% since 1940 [14]. Although localized pressures such as bycatch, maritime traffic, overfishing, and chemical and acoustic pollution continue to affect its distribution and abundance, the species is currently listed as Least Concern [15].

The only Ziphiidae, Cuvier’s beaked whale (*Ziphius cavirostris*), previously defined as Data Deficient [16], is now listed as Vulnerable [17]. Other regular Delphinidae species, such as the common dolphin (*Delphinus delphis*), Risso’s dolphin (*Grampus griseus*), and the pilot whale (*Globicephala melas*), are all declining in the Mediterranean area and are listed as Endangered [8].

Among the regular cetacean species in the Mediterranean, only two have a colossal size: the sperm whale (*Physeter macrocephalus*) and the fin whale (*Balaenoptera physalus*). The sperm whale is the largest of the odontocetes and feeds mainly on squid preyed upon at great depths. The fin whale is the only regular mysticete in the region and primarily feeds on plankton. The sperm whale was classified as Endangered in 2012 [18], and in 2021, its conservation status was reaffirmed under criterion C2a(ii), as data indicate that there are still fewer than 2500 mature individuals in the Mediterranean subpopulation [19]. Since the previous assessment [20], the fin whale’s Mediterranean subpopulation has continued to decline, reaching the threshold of less than 2500 mature individuals in a single population (48% of the total population being mature [21]), thus qualifying their upgrade from Vulnerable to Endangered under criterion C2a(ii) [22].

These assessments underscore the urgent need for species-specific and regionally tailored conservation strategies to address the varying levels of risk faced by Mediterranean marine mammal populations. The lack of basic distribution and ecological information about cetaceans can affect the conservation status of taxa, putting species or populations at some level of risk for potential long-term or short-term extinction, and can subsequently increase the difficulties associated with conservation planning [23,24,25,26,27,28,29]. The conservation status of marine mammals in the Mediterranean is adversely affected by epizootic outbreaks and a range of anthropogenic threats, such as fishery interactions, naval sonar operations, ship strikes, chemical pollution, and the ingestion of plastic debris [30].

The marine mammal survey is essential for monitoring population distributions and variations, which are constantly changing. These distributions can vary due to environmental changes, shifts in prey availability, or competition from other species, particularly humans [31,32]. At the same time, new and more effective research methods, techniques, and technologies are becoming available and applicable to these studies.

Scientific research employs various methods to census cetacean populations, each tailored to specific species, habitats, and research objectives. These methodologies are often combined to enhance accuracy and comprehensiveness. The primary technique used involves line-transect surveys. This approach is effective for large-scale assessments, especially when using ships or aircraft [33,34,35]. Another non-invasive method is photo-identification, capturing images of individual cetaceans’ unique physical features, such as dorsal fins or flukes, facilitating mark–recapture analyses to estimate population sizes and monitor movements [36,37], as in humpback whales (*Megaptera novaeangliae*) [33].

Moreover, Passive Acoustic Monitoring (PAM) employs underwater hydrophones to detect and record cetacean vocalizations [38,39,40]. This technique is advantageous for monitoring elusive species that inhabit deep waters or are active during nighttime when visual surveys are challenging [41,42]. By analyzing acoustic data, researchers can infer the presence and distribution, and, in some cases, estimate the relative abundance of cetacean populations [43].

Surveys can be conducted using genetic material too, collected through both invasive and non-invasive techniques [44,45,46]. The sampling method could be carried out through direct tissue sampling, for example, tissue biopsies using punch tools from sharks and rays [47,48,49], or with darts launched using air guns or crossbows to collect skin and blubber tissue samples from cetaceans [50,51]. These samples generally yield high-quality and large-quantity genetic material, and the metadata available has shown that biopsy wounds heal quickly in some species [51,52]. However, these sampling methods require direct contact with target individuals, which can trigger even lethal behavioral responses [53], and necessitate both training of investigators and application for collection permits before sampling [51,54].

Over the last few decades, there has been a surge of interest in non-invasive sources of DNA [55], ranging from sloughed skin from sperm whales [56,57] and humpback whales [58] to fecal plumes in dolphins [59] and mucus from manta rays, *Mobula birostris* [47]. Seawater filtration for environmental (e)DNA is also a rapidly expanding field with applications in cetology [58,59,60,61,62,63,64,65,66]. For cetaceans specifically, exhaled breath condensate, commonly referred to as ‘spout’ or ‘blow’, has been used as a non-invasive source of biological material over the last decade [67,68,69].

Additionally, genetic tools can be used on stranded marine mammals or their organ remains when morphological identification is extremely difficult or impossible due to a high degree of degradation and loss of many morphological features [70,71,72].

A wide variety of forensic tools designed specifically for cetacean species identification [73,74,75,76,77,78,79,80] are now available. Molecular markers, such as the random amplification of polymorphic DNA (RAPD), microsatellite, and mitochondrial DNA (mtDNA) genes, such as cytochrome b (CYTB) or cytochrome c oxidase I (COX-I), have been used to identify incidences of species trading of marine mammal species in the form of food products and stranded carcasses [24,74,75,81,82,83,84].

Recently, High-Resolution Melting analysis (HRM) has been introduced as a rapid method for genotyping known variants and for scanning for unknown variants [85]. This method has several notable advantages, such as the reduction in cross-contamination, speed of execution, and the fact that it does not require the handling of hazardous materials [86,87]. In addition, specimens can be distinguished by graph changes in the melting curves that are generated by HRM and are easily visualized [88]. HRM can reveal different melting temperatures, which can then be measured in real time, thereby reducing the subjective errors associated with human biases [89,90,91,92,93,94]. HRM analysis provides a precise, rapid, economical, and sensitive method for assessing genetic diversity, enhancing the accuracy of cetacean species-level identification, and offering a valid alternative to traditional PCR and sequencing approaches. Previously, HRM has been used with inter-simple sequence repeat (ISSR) markers for cetacean discrimination in sixteen species [70]. However, this method is not applicable to some Mediterranean species, such as the sperm whale.

Here, we propose a protocol based on HRM to identify four representative marine cetacean species that occur in the Mediterranean Sea: two of the most abundant Delphinidae, the coastal bottlenose dolphin and the pelagic striped dolphin, along with the two giants, the sperm whale and the fin whale. We designed a species-specific primer set, focusing on mtDNA D-loop long regions and the cytochrome b gene, that is able to discriminate species with high resolution in a single experiment.

The protocol was developed using four reference samples. The HRM curves obtained from the analyses were distinguishable for each of the four cetacean species analyzed, highlighting strong specificity and a clear separation. Subsequently, we successfully applied our HRM protocol to other DNA from unknown samples extracted from muscle and fecal plumes, correctly identifying the species.

Finally, this method can be applied in molecular taxonomy where morphological identification may be unfeasible due to specimen condition, in field activities such as non-invasive environmental collection, or during routine customs inspections and wildlife crime investigations, supporting legal proceedings under EU Regulation 1143/2014 and CITES enforcement protocols.

## 2. Results

The results provided insights into the genetic patterns of Mediterranean cetaceans based on mitochondrial DNA analysis. The regions selected for mtDNA analysis were cytochrome b and the D-loop, chosen for their optimal size for the HRM technique, which ranges from 80 to 220 base pairs. We tested a total of 21 mtDNA primers in combination to obtain a single mixture of four suitable primer pairs, which generated clear and reproducible melting fingerprints (Table S1).

In the final mix, each amplified DNA region had a unique sequence, and we analyzed the amplicon composition considering the % of each nucleotide (Adenine [A], Thymine [T], Cytosine [C], and Guanine [G]) and the GC content, which influences the melting temperature (Tm) during HRM reactions (Table 1).

**Figure 1:**
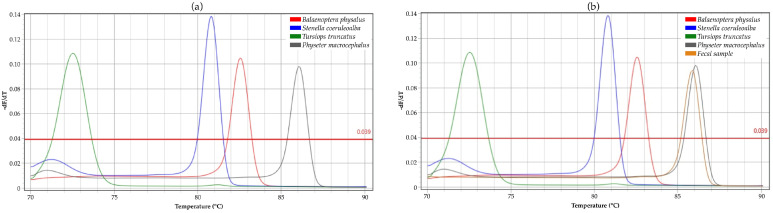
High-Resolution Melting curve analyses. (**a**) Melting profiles of positive controls with primer mix; (**b**) melting profiles of unknown fecal samples. Each color represents a species: *Balaenoptera physalus* in red, *Stenella coeruleoalba* in blue, *Tursiops truncatus* in green, *Physeter macrocephalus* in gray, and fecal sample in orange.

The protocol was tested on four reference samples, observing a clear difference in melting profiles (Figure 1a and Figure S1). Each sample was sequenced, and the species were confirmed by BLAST searches on the NCBI website with the highest identity scores (96–100% identity).

To verify primer specificity, primer pairs were selected following a thorough evaluation of both intraspecific and interspecific single-nucleotide polymorphisms (SNPs) to achieve greater accuracy and reliability in downstream molecular analyses (Table 2). Intraspecific SNPs were identified among individuals of the same species as the reference organism, while interspecific SNPs were detected by comparing the reference to closely related phylogenetic species (Table 2 and Table S1). This SNP-based comparative approach ensured that the primers exhibited high specificity toward the target species, minimizing nonspecific amplification.

Subsequently, the protocol was applied to all unknown samples, even DNA extracted from fecal samples (Figure 1b and Figure S2). The melting behavior of mtDNA fragments of each sample was predicted by analyzing the four reference samples. HRM analyses were successful in more than 95% of cases. The samples achieved a high-quality (HQ) score of over 90, confirming the reliability of the data. This high score made the data suitable for comparison with reference sequences in databases.

**Figure 2:**
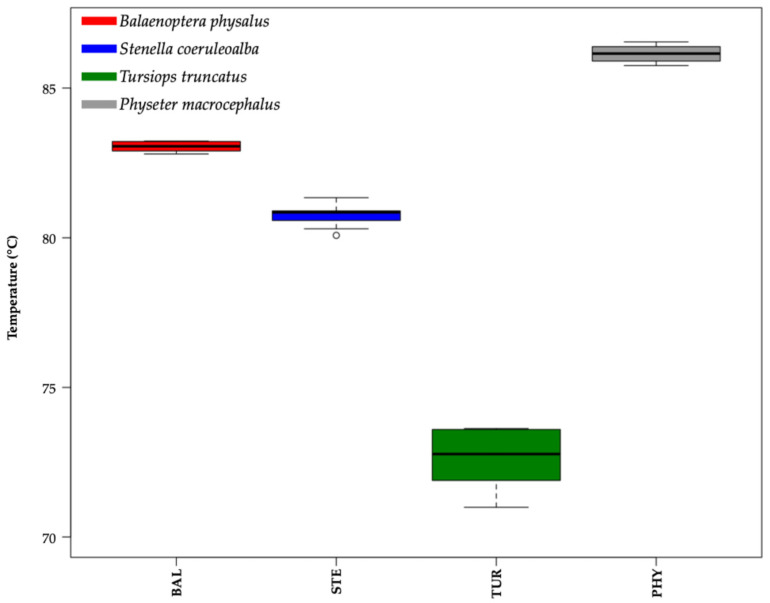
Box plot of melting temperature values (°C) of the main melting peaks (MMPs) of all samples analyzed. Delta temperature (ΔT) between the means of their main melting peaks (MMPs) are ΔT1 = 2.29 (*Balaenoptera physalus* vs. *Stenella coeruleoalba*); ΔT2 = 8.10 (*Stenella coeruleoalba* vs. *Tursiops truncatus*); and ΔT3 = 3.04 (*Balaenoptera physalus* vs. *Physeter macrocephalus*). One-way ANOVA test *p*-value < 0.05. Colors were used to distinguish different species: *Balaenoptera physalus* (BAL) in red, *Stenella coeruleoalba* (STE) in blue, *Tursiops truncatus* (TUR) in green, and *Physeter macrocephalus* (PHY) in gray.

The main melting peaks (MMPs) were 83.05 ± 0.18 °C for *Balaenoptera physalus*, 80.76 ± 0.28 °C for *Stenella coeruleoalba*, 72.66 ± 1.00 °C for *Tursiops truncatus*, and 86.08 ± 0.32 °C for *Physeter macrocephalus* (mean ± standard deviation; Figure 2). Moreover, the box plot performed considering the temperature values (°C) of the MMPs of all samples showed a clear aggregation of the data into four distinct groups, recognizable in different cetacean species (Figure 2, one-way ANOVA test *p*-value < 0.05).

The Sanger sequencing results showed an identity ranging from 96% to 100% and 100% coverage, indicating an optimal match between the obtained sequences and those in the reference databases, supporting the accuracy of the analysis.

## 3. Discussion

### 3.1. Applicability of HRM on Cetacean DNA Genotyping

Conservation management of animal species often involves complex and costly protocols, particularly when genetic analyses are included. Here, we propose a new protocol based on High-Resolution Melting, providing an efficient and reliable workflow for differentiating and identifying principal cetacean species in the Mediterranean Sea from DNA samples without having to sequence the majority of sample PCR products. In addition, we applied this technique for the first time to fecal samples of the sperm whale, demonstrating its versatility and applicability in a non-invasive genetic survey.

The portability and cost-effectiveness of this method make it particularly suitable for field applications, especially in remote areas or during rapid-response conservation interventions [96,97,98]. Its integration into surveillance protocols at customs or during marine patrols could enhance enforcement against illegal cetacean trade and trafficking [72,74,75,85,89,91,93,94,99]. HRM is based on the principle of a small yet definite shift in denaturation temperature due to nucleotide base variation. The amplified DNA is subjected to stepwise heating to obtain controlled denaturation of the amplicons. HRM is highly specific to the analyzed species, with a unique melting temperature, included in a heteroduplex assay. In this study, the difference in nucleotide composition was reinforced using a species-specific primer mix, allowing each species to have an amplicon that differs in fragment length, nucleotide variation, and GC content (Table 1). Typically, the amplicons should be shorter than 300 bp (with a maximum of 189 bp in this study), as longer fragments tend to produce less pronounced differences in melting curves between samples due to small nucleotide variations [100]. Other protocols use the same primers for different species, focusing on nucleotide sequence variation, which is reflected in different melting temperatures. In our case, we used amplicons that differed among species, thereby increasing sequence variations, which served as a good indicator of success in HRM analysis. The implementation of this method requires the use of a specific HRM machine, which can be used both in a standard laboratory and directly in the field. The use of a user-friendly and standardized protocol and the MIC qPCR Cycler (Bio Molecular Systems) ensures fast and highly accurate results, allowing for the simultaneous inspection of 48 specimens per run. Our protocol was developed on a compact and portable real-time PCR instrument bundled into a 2 kg cube, smaller and lighter than other available real-time PCR systems, making it ideal to use in the field and on a boat (as performed at the Stefano Mariani Laboratory in Liverpool, John Moores University in the UK [101]). Moreover, MIC is an instrument that utilizes a generation technology based on magnetic induction, capable of guaranteeing high and accurate performance and reproducing the same results not only across multiple runs but also across different instruments. This technology enables a high level of confidence in the identification of SNPs and insertion–deletion events, and maintains temperature uniformity in each well (±0.05). This technique, combined with species-specific primer design, enabled us to achieve a significant ΔT among the four analyzed species (Figure 1 and Figure 2).

The primers and protocols proposed in this study are also applicable in standard PCR, combined with Sanger sequencing, for species characterization. Our research demonstrates the effectiveness of HRM as a rapid and reliable method for species identification, which is crucial for stranding management [70,71,72]. Furthermore, we performed a validation during non-invasive collection on degraded samples, such as fecal ones. HRM analysis yielded high sensitivity and confidence intervals ≥ 90%, enabling successful species identification and supporting marine biodiversity monitoring.

Moreover, HRM analysis performed on MIC is a rapid, closed-tube, and comparatively inexpensive technique based on the fluorescent monitoring of DNA dissociation behavior when exposed to increasing temperatures. Molecular techniques like DNA barcoding and gene sequencing are valuable for obtaining accurate results from degraded or limited samples, such as stranded carcasses or illegal body parts involved in the bushmeat trade. These techniques may be the only way to identify cetacean species in such cases. Cetacean-specific primers were used to amplify mitochondrial DNA (mtDNA) regions, enabling precise species identification and genetic diversity analysis within cetacean communities. The use of mtDNA is particularly advantageous for degraded samples, as it is abundant and easier to characterize than nuclear DNA [70]. Mitochondrial DNA markers are often preferred over nuclear markers for species discrimination because mtDNA is present in many copies per cell. As a result, mtDNA is much easier to recover and amplify, especially when working with degraded, scarce, or low-quality samples, such as those obtained from stranded animals [102] or environmental samples [103,104,105].

Our findings align with previous studies, demonstrating the utility of HRM analysis in species identification, particularly in contexts where rapid, reliable, and cost-effective molecular tools are required [106]. This work bridges the gap between academic research and on-the-ground conservation, making it a significant addition to cetacean DNA genotyping.

HRM has been successfully applied to distinguish closely related taxa in various taxonomic groups, including mammals, birds, and fish [98,100,107], even on fecal samples [108,109]. However, unlike earlier protocols that relied on identical primers for multiple taxa—thus potentially limiting discriminatory power and increasing the risk of ambiguous melting profiles—our approach employed a species-specific primer design, tailored to maximize interspecific divergence in melting behavior. This strategy improves analytical resolution, enhances specificity, and minimizes the occurrence of cross-reactivity among non-target species [97,107].

HRM, unlike Sanger sequencing, is an unbiased qualitative assay that detects any mutation in the target gene region, allowing researchers to focus expensive analysis only on samples that show non-matching HRM profiles for barcode sequencing. In fact, HRM can be performed as an additional procedure following an initial PCR at a lower cost compared to direct Sanger sequencing. Finally, the cost of the necessary reagents to perform the analysis per sample was estimated at USD 3, considerably lower than the USD 10 for DNA barcoding sequencing and the USD 300 needed for whole-genome sequencing [96]. This protocol requires DNA extracted from reference samples, but tissues are obtainable from tissue banks, and in the future, the need for positive controls within laboratories may be reduced with increased implementation and data-sharing leading to the generation of online reference databases of HRM profiles from different real-time PCR platforms and laboratories, as well as machine learning algorithms that can be used to develop automated HRM classifications [97,98].

These results are crucial for the conservation of marine species and biodiversity management as they prevent misidentifications that could undermine conservation efforts. This advancement represents a meaningful contribution to molecular taxonomy, especially for cetaceans, where morphological identification may be unfeasible due to the condition of the specimen or the context of sample collection. Molecular approaches can contribute to understanding population dynamics and informing protection measures.

### 3.2. Application in Wildlife Forensics

Finally, the illegal trade in cetacean products remains a conservation and enforcement challenge, despite the international regulatory framework established by the Convention on International Trade in Endangered Species of Wild Fauna and Flora (CITES, 1973). Countries such as Japan, Norway, and Iceland continue to exploit legal exemptions (e.g., for scientific research or under objection clauses) to engage in whaling activities, while a parallel black market persists for products including cetacean meat, oil, bones, teeth, and skin [75,110]. These products are trafficked both within and beyond national borders, often under false labeling or concealed among other seafood items. Within the European context, Italy has been identified as both a transit and end-market country. Official data from the Italian Forestry Corps (Corpo Forestale dello Stato) and customs agencies, as well as reports by NGOs such as Traffic and Sea Shepherd, document over 30 confirmed seizures of cetacean derivatives between 2010 and 2020, including illegal imports of meat from Asia and the sale of souvenirs derived from whale bones and teeth in tourist areas [111].

Estimates suggest that the black market value of cetacean-derived products in Italy may exceed EUR 2 million per year, although the true scale is difficult to quantify [112]. In addition to its biodiversity impact, illegal consumption of cetacean meat poses serious public health risks, particularly due to the high levels of mercury and persistent organic pollutants typically found in whale tissues [113]. These concerns underline the importance of integrating molecular forensic tools such as DNA barcoding, real-time PCR, and HRM analysis [114] into routine customs inspections and wildlife crime investigations in order to improve traceability, enable species-level identification, and support legal proceedings under EU Regulation 1143/2014 and CITES enforcement protocols [98,99,115]. Moreover, further research could focus on other cetacean species inhabiting the Mediterranean region and from other parts of the world, as well as on environmental DNA (eDNA) analysis [104], which enables monitoring of species distribution without direct observation or capture [116,117].

## 4. Materials and Methods

### 4.1. Sampling and DNA Extraction

A total of 28 muscle tissue samples were collected from four cetacean species in the Mediterranean Sea: the bottlenose dolphin (*Tursiops truncatus*; N = 5), the striped dolphin (*Stenella coeruleoalba*; N = 15), the sperm whale (*Physeter macrocephalus*; N = 4), and the fin whale (*Balaenoptera physalus*; N = 4).

Biological tissues from marine mammals were provided by the Experimental Zooprophylactic Institute of Southern Italy and the Department of Life and Environmental Sciences at the Polytechnic University of the Marche (CITES n° 4814/2019/PAB). Total genomic DNA was extracted from 25 mg of tissue using a DNeasy Blood & Tissue Kit (QIAGEN GmbH, Hilden, Germany), following the manufacturer’s instructions. The integrity of the extracted DNA was evaluated by 1% agarose gel electrophoresis, while concentration and purity were measured using a NanoDrop 2000 spectrophotometer (Thermo Fisher Scientific™, Wilmington, DE, USA).

### 4.2. Coprological Sampling and Analyses

**Figure 3:**
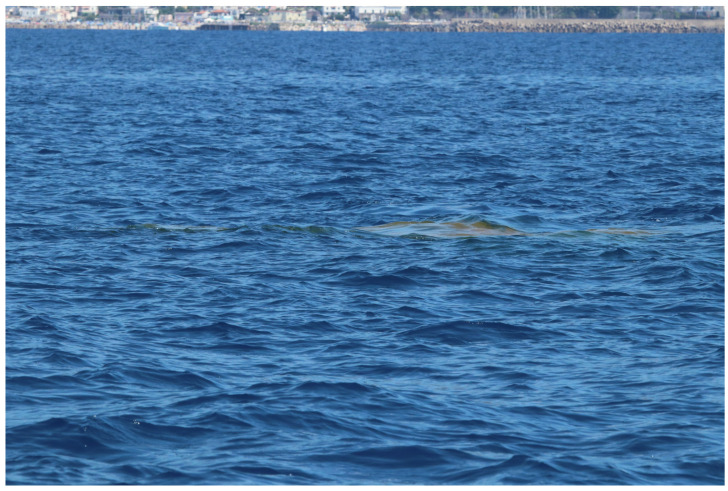
Feces sampling in the wild (photos by Angelo Miragliuolo—Oceanomare Delphis).

The “Waters of Ischia and Ventotene” Important Marine Mammal Area, IMMA, is well known as a diversity hotspot of pelagic fish, sea birds, and cetaceans [118,119,120] in the central Mediterranean Sea. In the framework of a research project on the ecology of whales conducted aboard Jean Gab, a 17.70 m oak cutter, two fecal samples were collected from sperm whales (Figure 3). Floating feces were collected from individual whales, avoiding direct contact with animals, using a fine nylon mesh net. Fecal samples were immediately placed in sterile tubes, labeled for identification, and stored at −20 °C for further analysis. Genomic DNA was extracted from each fecal sample of approximately 200 mg by using a QIAamp DNA Stool Mini Kit (Qiagen, Hilden, Germany) according to the manufacturer’s instructions and then eluted in 200 μL TE buffer.

### 4.3. Primer Design

The Mediterranean Sea is recognized as a biodiversity hotspot for cetaceans, hosting a diverse community of resident and migratory species, despite the increasing anthropogenic pressures threatening their conservation [1,30,121,122,123]. We focused on the four representative marine cetacean species that occur in the Mediterranean Sea: two of the most abundant Delphinidae, the coastal bottlenose dolphin and the pelagic striped dolphin, along with the two giants, the sperm whale and the fin whale.

For species identification, we designed highly specific primers to provide variability among species and individuals within each species (Table S1).

We focused on two mitochondrial genomic regions that are more suitable for this type of study than nuclear DNA due to their higher copy number, circular structure, and lower susceptibility to degradation. One region is the cytochrome b gene, which is highly conserved but exhibits significant genetic variability, making it a valuable tool in molecular evolution studies to trace phylogenetic relationships between species. The other region is the D-loop, one of the most variable regions, less subject to selection pressure, allowing it to accumulate polymorphisms more rapidly. To achieve better differentiation of the melting curves, primer pairs were designed to amplify distinct loci in these two mitochondrial regions.

Primers were designed using Geneious Prime software (version 2024) based on publicly available mitochondrial sequences of marine mammals and validated using Primer-BLAST (https://www.ncbi.nlm.nih.gov/tools/primer-blast/, accessed on 30 July 2025) to ensure species specificity and amplicon size discrimination.

A total of 21 primer pairs were selected for the four cetacean species (Table S1), each designed to amplify sequences of different lengths, thereby ensuring that PCR products generate clear and easily distinguishable bands.

**Figure 4:**
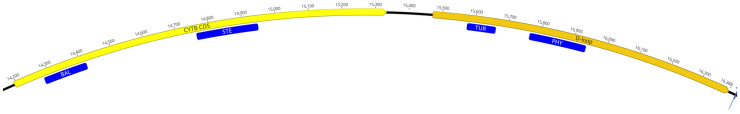
Scheme of mitochondrial DNA (mt-DNA) region targeted by primers used for HRM; TUR—bottlenose dolphin (*Tursiops truncatus*); STE—striped dolphin (*Stenella coeruleoalba*); PHY—sperm whale (*Physeter macrocephalus*); and BAL—fin whale (*Balaenoptera physalus*).

For each species, DNA templates were tested first using species-specific primers, but subsequently, we tested different primer mixes targeting all four species. We selected four of these primer pairs, which provided species-specific bands and were suitable for further HRM analysis (Figure 4 and Table 3).

We tested the feasibility of the primer sets through PCR in a total volume of 20 μL, using 100 ng of DNA, 10 μL of Platinum™ Hot Start PCR Master Mix (2×) (Invitrogen™, Thermo Fisher Scientific, Waltham, MA, USA), and 10 μM of primer mix.

PCR conditions were set with an initial denaturation step at 94 °C for 2 min, followed by 35 cycles at 94 °C for 15 s, 60 °C for 15 s, and 68 °C for 15 s.

PCR products were sequenced (Eurofins Genomics, https://eurofinsgenomics.eu/, accessed on 30 July 2025, Ebersberg bei München, Germany) and the resulting chromatograms were aligned and compared with D-loop and cytochrome b sequences available on GenBank.

### 4.4. High-Resolution Melting (HRM)

HRM was performed on an MIC qPCR Cycler (Bio Molecular System, Brisbane, Queensland, Australia) according to the manufacturer’s protocol. We evaluated the effectiveness of HRM analysis in a total volume of 20 μL, using 100 ng of DNA, 10 μL of Clara^®^ HRM Mix (2×) (PCR Biosystems Ltd., Aztec House, London, UK), and 10 μM of primer mix.

Cycling conditions consisted of an initial denaturation step of 2 min at 95 °C, followed by 45 cycles of 5 s at 95 °C and 30 s at 64 °C. The melting step was set at 95 °C for 60 s, followed by 55 °C for 60 s, and an increase in temperature from 55 °C to 95 °C with 0.025 °C increments of 2 s at each temperature.

The results obtained from the HRM reactions were analyzed using the HRM data interpretation software BMS Workbench v. 1.4.2 (Bio Molecular System) and the unknown samples were assigned to their corresponding cetacean species based on the obtained melting profile, comparing it with the melting curves of the reference samples previously analyzed.

### 4.5. Quality Control and Validation Procedures

To ensure the reliability and reproducibility of the experimental procedures, a series of control measures were implemented throughout the study. In particular, species assignment of tissues, performed using morphological data, was confirmed by Sanger sequencing. Fecal samples were collected following standardized, sterile procedures to avoid contamination. Subsequently, during DNA extraction, PCR amplification, and HRM analysis, negative controls were included to monitor for possible contamination of reagents. Positive controls for each target species were included in every PCR and HRM run to confirm the specificity and reproducibility of the amplification and melting profiles. HRM analyses were performed independently in duplicate to verify the stability of the melting profiles. Only profiles showing consistent replicates and matching with positive controls were considered valid. Finally, sequencing was used to validate the HRM results.

## 5. Conclusions

The HRM analysis developed in this study offers a reliable, rapid, and cost-effective alternative to traditional PCR and sequencing methods for accurate species-level identification and classification. Our approach focuses on four marine mammals: two abundant species of the Delphinidae family and the two largest whale species. The method was validated on both tissue and environmental samples, making it ideal for non-invasive sampling during field activity. Future applications should extend to all regular Mediterranean species as well as cetacean species from around the world. The development of genetic analysis on environmental samples could overcome the adverse effects of invasive sampling on the welfare of these animals, as tissue biopsies may trigger behavioral modification and even lethal responses. Moreover, boat-based surveys can generate chemical and acoustic noise; therefore, using silent and low-impact vessels, such as the Jean Gab oak cutter, could minimize disturbance and ensure that conservation research aligns with animal welfare principles [124,125].

## Figures and Tables

**Table 1:** Amplicon composition for the primer pairs selected for each cetacean species.

Species	Primers	Size (bp)	%A	%C	%G	%T	%GC
*Balaenoptera physalus*	B1F-B1R	133	29.3	33.9	13.5	23.2	47.7
*Stenella coeruleoalba*	S1F-S1R	189	32.9	32.9	10.2	24.7	43.1
*Tursiops truncatus*	T1F-T11R	86	26.5	17.4	11.7	44.4	29.1
*Physeter macrocephalus*	P1F-P1R	175	21.7	30.9	22.7	24.8	53.6

**Table 2:** Number of intraspecific and interspecific single-nucleotide polymorphisms (SNPs). Interspecific comparison was performed with species in the same geographical distribution area or that were phylogenetically related [95]. MED = Mediterranean Sea; CAA = contiguous Atlantic area.

Species	Primers Name	SNPsIntraspecific	SNPsInterspecific
*Balaenoptera physalus*	B1F-B1R	1	8 *B. musculus* (north of the Antarctic Convergence, most abundant in waters off Australia, Madagascar, and New Zealand); 9 *B. borealis* (MED, CAA); 10 *B. acutorostrata* (MED, CAA)
*Stenella* *coeruleoalba*	S1F-S1R	4	11 *S. longirostris* (Pacific, Atlantic, and Indian Oceans, including the Persian Gulf and the Red Sea; they do not occur in the Mediterranean Sea); 1 *S. clymene* (northwestern Atlantic, Gulf of Mexico, the Caribbean, and along the coasts of West Africa and southern Brazil); 6 *Delphinus delphis* (MED, CAA); 12 *T. truncatus* (MED, CAA)
*Tursiops* *truncatus*	T1F-T11R	2	3 *Tursiops aduncus* (Indo-pacific); 3 *S. coeruleoalba* (MED, CAA)
*Physeter* *macrocephalus*	P1F-P1R	0	59 *Kogia sima* (MED); 57 *K. breviceps* (Atlantic, Pacific, and Indian Oceans)

**Table 3:** Primers selected for HRM analysis in this study.

Species	Primer Name	Sequence 5′-3′	Size (bp)	Region
*Balaenoptera* *physalus*	B1F	TGGAACTTCGGCTCCCTACT	133	Cytochrome b
	B1R	AATTCACGTCTCGGCAGATG		
*Stenella* *coeruleoalba*	S1F	CACAGCATTAGCAGCCGTTC	189	Cytochrome b
	S1R	CCTAGTAGGTCGGGGGTGAA		
*Tursiops* *truncatus*	T1F	TGCGCATGCTAATATTTAGTCTCT	86	D-loop
	T11R	TCGTATGGAAAATAAATGAATGCACAA		
*Physeter* *macrocephalus*	P1F	TGAGCTCTCGGATCAGACCA	175	D-loop
	P1R	GCAGGTGCCTCGAGTTATGA		

## Data Availability

The original contributions presented in this study are included in the article. Further inquiries can be directed to the corresponding author. The data presented in this study are openly available in Zenodo https://zenodo.org/records/15848426 (accessed on 30 July 2025) and in GenBank (NCBI https://www.ncbi.nlm.nih.gov/genbank/, accessed on 30 July 2025) at the reference numbers listed in Table S2.

## Supplementary Materials

The following supporting information can be downloaded at https://www.mdpi.com/article/10.3390/ijms26157517/s1.
