# Supplementary material for: When DNA Tells the Tale: High-Resolution Melting as a Forensic Tool for Mediterranean Cetacean Identification
Source: Int J Mol Sci. 2025 Aug 4;26(15):7517. doi: 10.3390/ijms26157517 (PMC12347690; doi:10.3390/ijms26157517)

Table S1. Primers designed in this study.

Species	Primer Name	Sequence 5′-3′	Region
Balaenoptera physalus	B1F	TGGAACTTCGGCTCCCTACT	Cytochrome b
	B1R	AATTCACGTCTCGGCAGATG	
	B2F	CTAGGCGGAGTCTTAGCCCT	
	B2R	AGGACTCAGAACAAGAACTGGC	
	B4F	GCCAGTTCTTGTTCTGAGTCCT	
	B4R	GGGGTGTTCTACTGGTTGGC	
Stenella coeruleoalba	S1F	CACAGCATTAGCAGCCGTTC	Cytochrome b
	S1R	CCTAGTAGGTCGGGGGTGAA	
	S2F	AATTGGAGGCCAACCCGTAG	
	S2R	AGGCCGGCTGTTGGTATTAG	
	S8F	CAGTCATGGCCACTGCATTC	
	S8R	CGGCTGCTAATGCTGTGATG	
Tursiops truncatus	T1F	TGCGCATGCTAATATTTAGTCTCT	D-loop
	T1R	TTCACGCGGCATGGTGATTA	
	T2F	ACAACACCACAGTACTACGTCA	
	T2R	AGCTCGTGATCTAATGGAGTGA	
	T11R	TCGTATGGAAAATAAATGAATGCACAA	
Physeter macrocephalus	P1F	TGAGCTCTCGGATCAGACCA	D-loop
	P1R	GCAGGTGCCTCGAGTTATGA	
	P5F	TCAGGCCCATTCCTCGAAAG	
	P5R	AAAGCTGGAACTAGCGCAGG	

Table S2. Sequences used to compare amplified regions among species.

Species Name	Accession Number	mtDNA Region
Balaenoptera physalus	NC_001321	Cytochrome b
Balaenoptera physalus	HM034293	Cytochrome b
Balaenoptera physalus	KC572709	Cytochrome b
Balaenoptera physalus	KC572714	Cytochrome b
Balaenoptera physalus	KC572828	Cytochrome b
Balaenoptera physalus	KC572829	Cytochrome b
Balaenoptera physalus	KC572860	Cytochrome b
Balaenoptera physalus	MF409243	Cytochrome b
Balaenoptera physalus	MT410921	Cytochrome b
Balaenoptera physalus	OR146987	Cytochrome b
Balaenoptera acutorostrata	AJ554054	Cytochrome b
Balaenoptera acutorostrata	AP006468	Cytochrome b
Balaenoptera acutorostrata	NC_005271	Cytochrome b
Balaenoptera borealis	AP006470	Cytochrome b
Balaenoptera borealis	MF409248	Cytochrome b
Balaenoptera borealis	NC_006929	Cytochrome b
Balaenoptera musculus	EF057442	Cytochrome b
Balaenoptera musculus	OQ865152	Cytochrome b
Balaenoptera musculus	ON257884	Cytochrome b
Stenella coeruleoalba	AF084081	Cytochrome b
Stenella coeruleoalba	AF084082	Cytochrome b
Stenella coeruleoalba	DQ466016	Cytochrome b
Stenella coeruleoalba	DQ466018	Cytochrome b
Stenella coeruleoalba	DQ466023	Cytochrome b
Stenella coeruleoalba	EF090637	Cytochrome b
Stenella coeruleoalba	MT410956	Cytochrome b
Stenella coeruleoalba	LT971412	Cytochrome b
Stenella coeruleoalba	DQ466020	Cytochrome b
Stenella coeruleoalba	ON959817	Cytochrome b
Stenella longirostris	30686051	Cytochrome b
Stenella longirostris	KX857355	Cytochrome b
Stenella longirostris	X56292	Cytochrome b
Stenella clymene	OL5505400	Cytochrome b
Stenella clymene	70614779	Cytochrome b
Stenella clymene	NC_060611	Cytochrome b
Delphinus delphi	MH00036512	Cytochrome b
Delphinus delphi	MT410915	Cytochrome b
Delphinus delphi	NC_036415	Cytochrome b
Tursiops truncatus	EU557093	Cytochrome b
Tursiops truncatus	OR120226	Cytochrome b
Tursiops truncatus	OR120227	Cytochrome b
Physeter macrocephalus	KU891337	D-loop
Physeter macrocephalus	KU891342	D-loop
Physeter macrocephalus	KU891344	D-loop
Physeter macrocephalus	KU891345	D-loop
Physeter macrocephalus	KU891354	D-loop
Physeter macrocephalus	KU891365	D-loop
Physeter macrocephalus	KU891370	D-loop
Physeter macrocephalus	KU891389	D-loop
Physeter macrocephalus	KU891391	D-loop
Physeter macrocephalus	ON725079	D-loop

Kogia breviceps	AJ554055	D-loop
Kogia brevicpes	NC_005272	D-loop
Kogia breviceps	AB571995	D-loop
Kogia sima	AB571997	D-loop
Kogia sima	AB571998	D-loop
Kogia sima	OQ736582	D-loop
Tursiops truncatus	AY963607	D-loop
Tursiops truncatus	AY963612	D-loop
Tursiops truncatus	AY963613	D-loop
Tursiops truncatus	AY963614	D-loop
Tursiops truncatus	AY963615	D-loop
Tursiops truncatus	AY963616	D-loop
Tursiops truncatus	DQ073641	D-loop
Tursiops truncatus	DQ073645	D-loop
Tursiops truncatus	DQ073663	D-loop
Tursiops truncatus	DQ073682	D-loop
Tursiops aduncus	MW118260	D-loop
Tursiops aduncus	MW118159	D-loop
Tursiops aduncus	MH733901	D-loop
Stenella coeruleoalba	EU5570972	D-loop
Stenella coeruleoalba	NC_012053	D-loop
Stenella coeruleoalba	EU557097	D-loop

Figure S1. HRM difference plot based on the analysis of 4 species. (**a**) Distinct normalized HRM profiles are represented as percent fluorescence changing in fluorescence units with increasing temperatures (dF/dT). (**b**) Difference graph of fluorescence. *Balaenoptera physalus* was used as a reference (red line in this graph) and the fluorescence of the samples minus the fluorescence of the reference was calculated (vertical axis). This graph is used to better visualize the differences between individual melting curves.

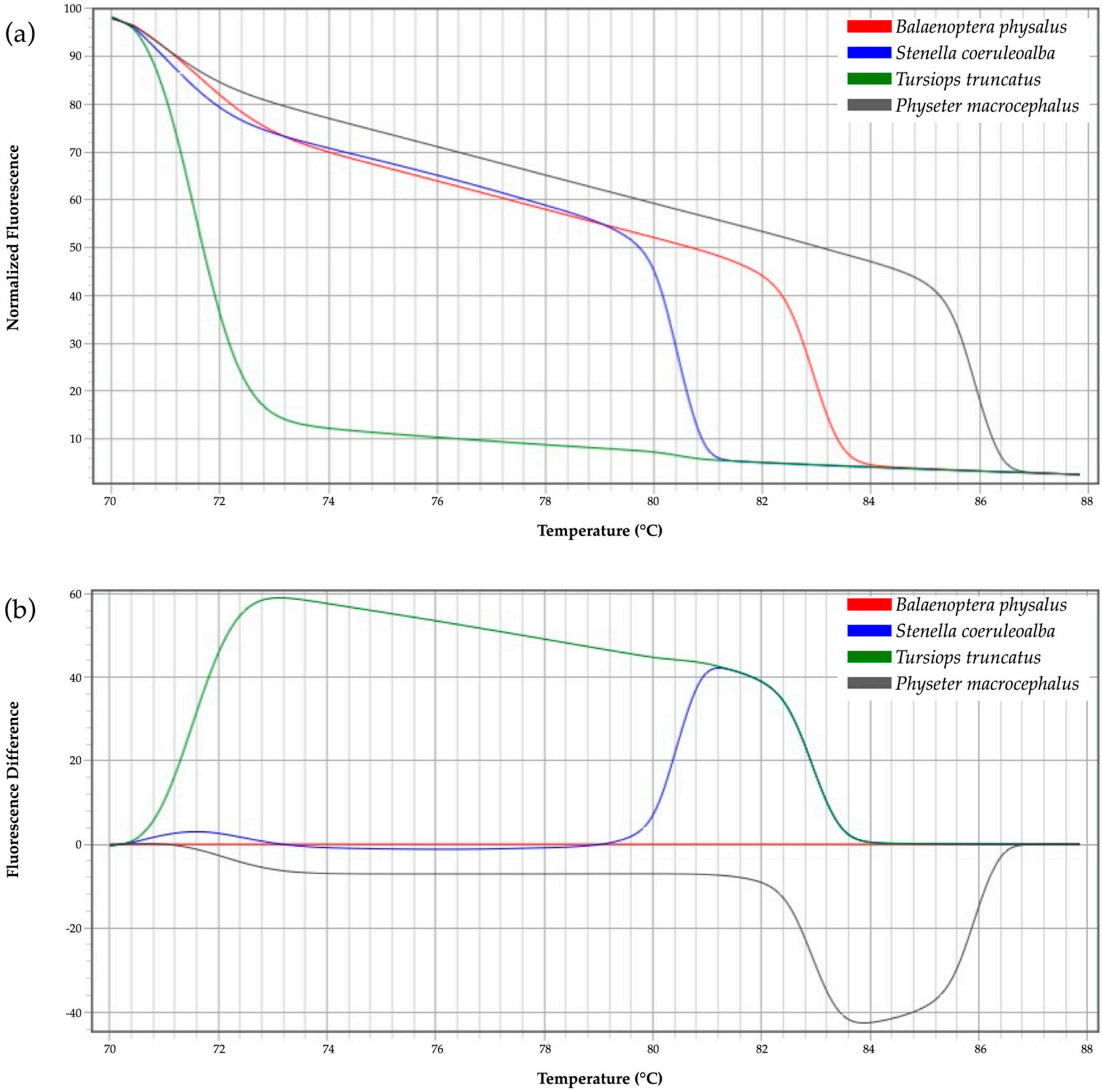

Figure S2. Melting curves derived from unknown DNA samples and reference .

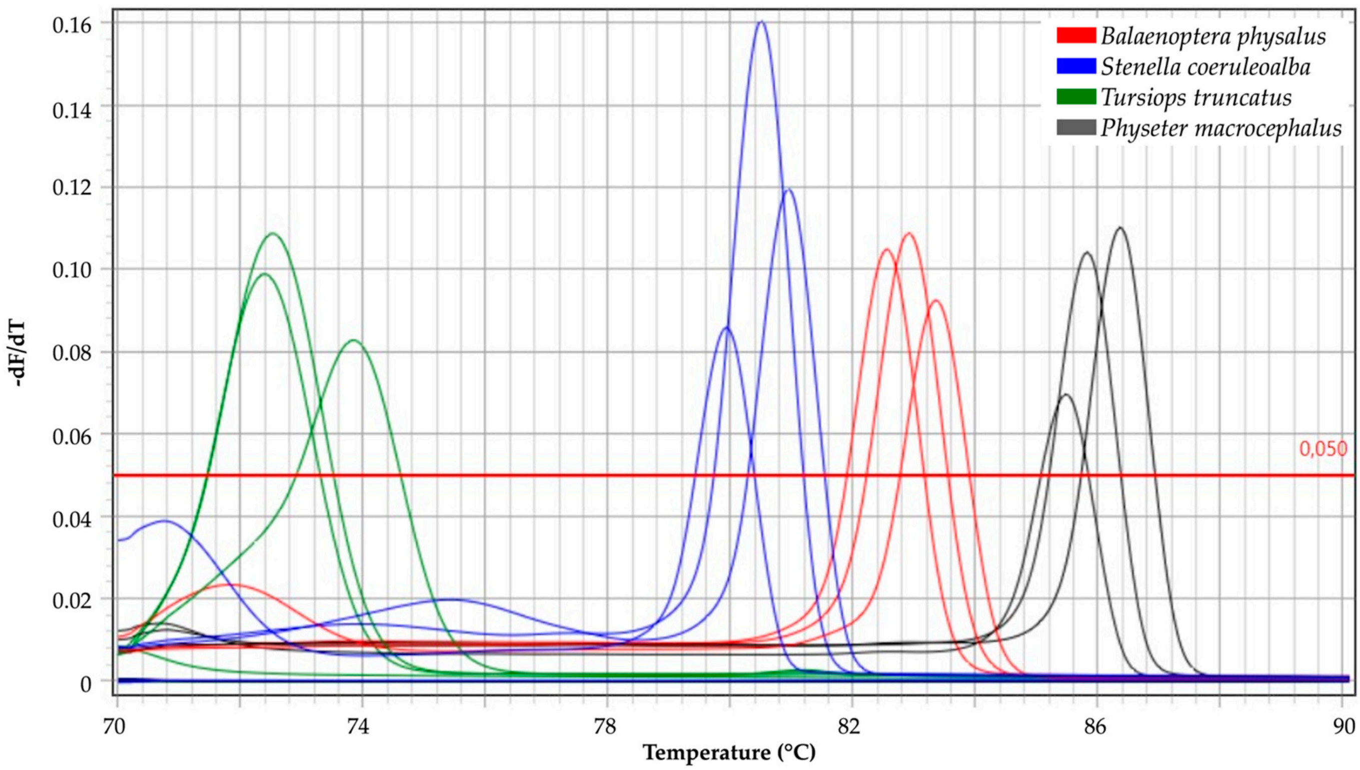

Supplement: Supplementary file 1 [file ijms-26-07517-s001.zip › ijms-3787904-supplementary.pdf]
